# Assessment of landslide behaviour in colluvium deposit at Doi Chang, Thailand

**DOI:** 10.1038/s41598-021-02363-3

**Published:** 2021-11-25

**Authors:** Suttisak Soralump, Avishek Shrestha, Worawat Thowiwat, Ramatre Sukjaroen, Thapthai Chaithong, Sirisart Yangsanphu, Abhishek Koirala, Apiniti Jotisankasa

**Affiliations:** 1 Geotechnical Engineering Division, Department of Civil Engineering, Faculty of Engineering, Kasetsart University, Bangkok, 10900 Thailand; 2 Geotechnical Engineering Research and Development Center, Kasetsart University, Bangkok, 10900 Thailand

**Keywords:** Engineering, Civil engineering

## Abstract

The paper presents the case study of the recurrent slope movement in colluvium deposits at Doi Chang, Thailand. A thorough site investigation confirmed the slope movement rate corresponding to slow creep during dry season, while in the rainy season, its velocity remarkably increased. Despite frequent repair, the movement rate was sufficient to result in the recurrent damage of infrastructures like roads and buildings, causing economic loss and public concerns. Furthermore, surface mapping revealed that the hill's topography led to the concentration of flowing water in a particular area. This resulted in a high level of groundwater table, especially during the rainy season. The inclinometer installed in that area suggested an average movement rate of 20.5 mm/month in the wet season. In contrast, during the dry season, it was limited within 2 mm/month, indicating that the increase in the rate of slope movement in the colluvium deposit was primarily due to the rising groundwater table. Field and laboratory tests were conducted to determine the properties of the colluvium deposit. Landslide susceptibility assessment was performed using infinite slope model and later integrated with GIS to evaluate the factor of safety (FS) over a large area. The FS decreased below 1 when the groundwater level rose to 0.3 cm below the ground surface, and using GIS, based on infinite slope model, the potential risk zone were delineated.

## Introduction

The exploration of landslide behaviour in the scientific community dates back to the first half of the twentieth century^1,2^. Many studies have been conducted on this topic from the beginning of the 1960s^3–6^. However, it still remains a significant challenge in natural hazard risk mitigation^7^ and it has been suggested that landslide behaviour prediction is an area of applied science that has not been well developed^8^. But even so, there have been cases worldwide where monitoring of the landslides coupled with forewarning has prevented loss of life and property^9^. This paper intends to present the adopted site investigation techniques and instrumentations to determine the cause and assess the landslide movement in the colluvium deposit at Doi Chang, Thailand.

Lying in a hilly region of northern Thailand, Doi Chang has received a geographical indication (GI) tag for the production of its high-quality coffee beans. Therefore, the place is of significant value to the local people and the Thai government for the revenue it generates. The settlement in Doi Chang is therefore growing, but the locals reported the recurrent damage of the infrastructures like walls, buildings, and roads in the area. The area's topography is built on a slight slope of 10 degrees, and therefore the slope movement at the first instance was unexpected. A thorough site investigation, including in-situ tests like standard penetration tests (SPT) and electrical resistivity tests (ERT), indicated a thick deposit underlying layer of colluvium materials.

Colluvium materials consist of soil, and rock debris with a heterogeneous mixture of grain sizes varying from large boulders to clay particle at the base of hill slopes. Normally deposited at the base of hillslopes as loose, unconsolidated sediments, colluvium is often of marginal stability and disturbance to it can cause recurrent downward movement. Generally, in normal conditions, the factor of safety (FS) is near 1.0–1.2 for colluvium deposits^10^. However, as witnessed in Doi Chang, FS less than unity may not always lead to dramatic slope failure in colluvium with a gentle slope. Rather the resulting slow downward movement of colluvial slope are associated with cracks in the buildings and road surfaces, which require frequent maintenance leading to public concerns^11^. Such damages were more pronounced in Doi Chang, especially after the rainy season.

Measurement of slope mass movement that integrates hydrological (i.e. rainfall or groundwater level) and geotechnical data (i.e. soil type or depth of slip plane) improved the knowledge of the mechanism of slope mass movement and slope stabilization^12,13^. A variety of techniques have been used to monitor and interpret the patterns of slope movement. In general, techniques like extensometers and inclinometers can collect data on the movement patterns for a small area, but these techniques have difficulty interpreting data on a larger scale^14^. For the larger area, photogrammetry and InSAR (Interferometric Synthetic Aperture Radar) can monitor surface displacements, but these techniques lack in terms of temporal resolution, and significant movement events are rarely captured in detail^15^. The rate and direction of slope mass movement can be determined using the relative position of the Global Positioning System (GPS) at centimeter or millimeter accuracy.

Similar innovation has been used in the present study to assess the landslide behaviour in the colluvium deposit resting at the middle portion of the hill slope in Doi Chang, Thailand. Use of the inclinometer, observation well, rain gauge station, and GPS survey has been undertaken to explore the rate and cause of slope movement. The combination of such instruments aims at capturing the movement pattern at local as well as larger areas. Furthermore, through the data obtained from the recording instruments, an attempt has been made to find the relationship between the geomorphological setting and hydrological characteristics with the slope mass movement occurring in the area. The results from this study have been used further by the officials at the ministry level to set up proper mitigation plans.

## Characteristics of the studied area

The study area is located at Doi Chang village, Vavee sub-district, Mae Suai District, Chiang Rai province, Thailand. The province includes Mae Ko complex that consists meta-volcanic rocks, which was mapped as Silurian-Devonian metamorphic rocks. The detailed investigation found basaltic andesite with some schist and phyllite rocks. The altitude of the area varies from 1000–1800 m above sea level (m.a.s.l.) and the slope ranges from 0–20 degrees.

**Figure. 1:**
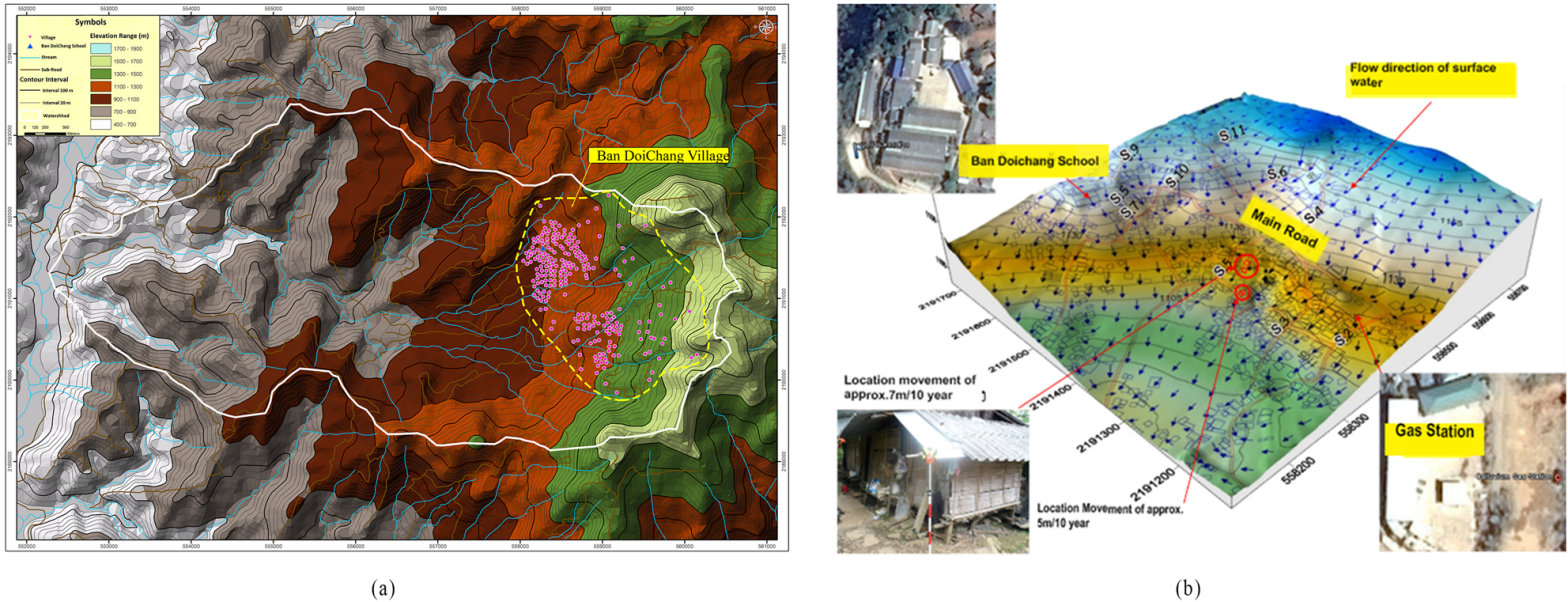
(**a**) Contour map of the study area along with settlements (**b**) 3D topography showing the direction of flow of surface water.

As coffee beans are produced and exported, the settlement and the business in this area are growing yearly. The settlement is concentrated mainly at the middle portion of the slope (Fig. 1), where the locals had been reporting recurrent damages to the infrastructures. The area's topography for this portion suggests a gentle slope of 10 degrees and considering the hill's steepness alone; its movement was initially thought unlikely to cause the reported damages.

During the field visit and survey, cracks were observed on the wall and fences. Similarly, longitudinal cracks were observed on the streets, and soil surface rupture was noticed in the village. The total number of housing units during the survey in the studied area was 407. Also, the total number of people living in the area was found to be around 6000. 7% of the total houses were categorized under severe damage, 35% under slight damage, 13% under moderate damages, while 45% of the houses reported no damage at all. Such categorization of the buildings was done on the basis of the extent of visible cracks^16^. It was noticed that houses categorized under severe and moderate damages fall mainly within a specific geographic location.

Using contouring software (Surfer), the 3D topography of the area was developed (Fig. 1b). School and gas station have been portrayed as prominent landmarks of the region. The flow direction of the surface water was analyzed using the hydrology tool of ArcGIS. It was found that the topography of the area was built in a way that led to the concentration of water in certain areas. The flow lines indicate the direction of water flow which appeared to converge in the area where the major and moderate damages of the houses were reported.

## Instrumentation and movement behaviour

In-situ ground-based monitoring techniques have been deployed to observe the possible slope displacement and measure the changes in attributes of landslide triggering factors like rainfall. This landslide monitoring method can be grouped into three categories: monitoring of rainfall, slope displacement, and hydrogeological and mechanical properties in soil^17^.

**Figure. 2:**
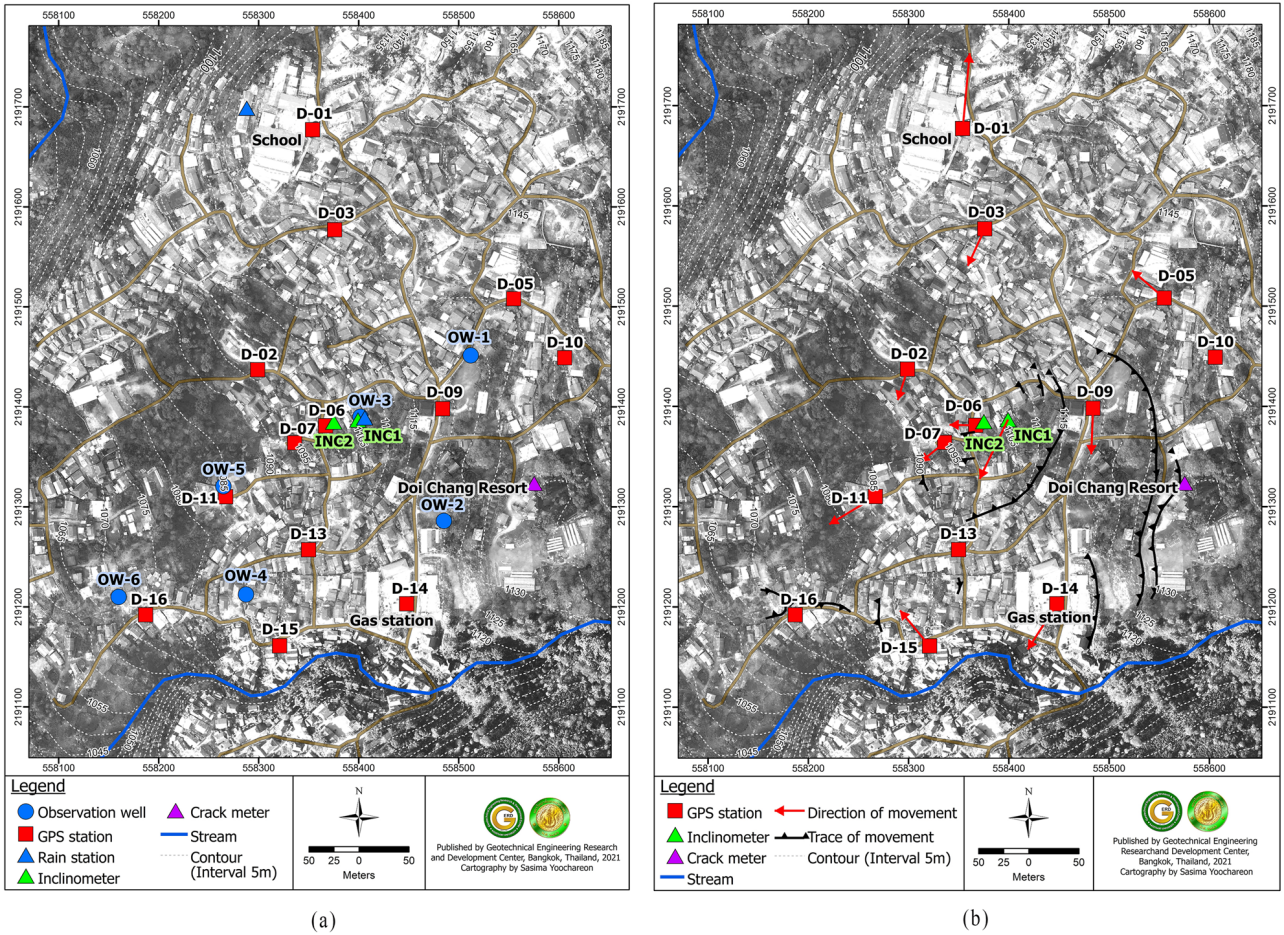
(**a**) Instrumentation at the study site (**b**) Direction of the slope movement.

Likewise, various instruments like rain gauge stations, observation wells, slope inclinometer^18^, temporary benchmarks were installed (Fig. 2a). The groundwater level and rainfall data were measured from 3rd March 2016–6th June 2021 through the observation wells (OW1–OW6) and rain gauge installed near the OW3 respectively. Periodic lateral displacement was measured using the inclinometer INC 1, beginning from March 2016 to Feb of 2017 after which it exceeded its capacity. After that, another inclinometer (INC 2) was installed about 25 m away from INC 1, and it recorded the lateral displacement for 2020/21. Similarly, five official geodetic marks installed by Royal Irrigation Department of Thailand were used and 12 temporary benchmarks were projected from it. These GPS stations were spread over the large area mainly to determine the direction of the movement, as during the rainy season it failed to provide data due to lack of signal connectivity with the satellites.

From the measurement of movement of GPS station, the range of slope mass movement was found to be 0.66 to 3.8 mm per month and the direction of the movement was southwest of the village (Fig. 2b) which formed the downslope of the area. However, it was noted that the GPS movement readings were taken in a relatively small period. The reading were taken in the dry season only, as during the wet season the weather affected the satellite reading of GPS.

**Figure. 3:**
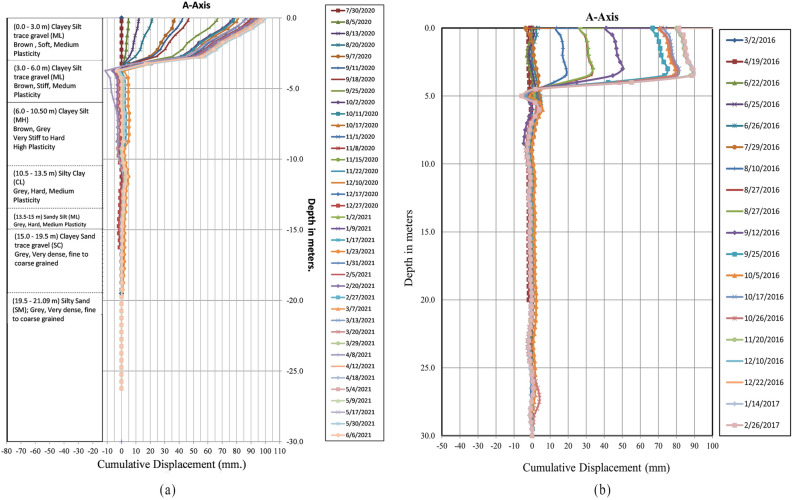
(**a**) Orientation of inclinometer in 2016 (**b**) Observation of inclinometer in 2020/2021.

The readings obtained from two inclinometers, INC 1 and INC 2 are shown in Fig. 3. The readings taken from the inclinometer are for the different periods and place (about 25 m away from each other). However, both inclinometers demonstrate shear plane at a depth of 3–5 m from the ground.

In 2016/17 the total accumulated movement was about 92 mm with an average movement rate of 19.45 mm/month. The maximum movement rate of 66 mm/month was observed during the month of September. The inclinometer exceeded its capacity and hence it needed to be reinstalled. Similarly, from the records of 2020/21, the prime movement of the slope was observed from July to November which was a rainy season, and a cumulative movement of more than 80 mm was detected. The average rate of movement was 20.45 mm/month, with a maximum movement rate of 40 mm/month at a depth of 3.5 m in September.

A measure of landslide risks requires measurement of velocity and the velocity of slope movement has been categorized into seven classes, 1–7, 1 being extremely slow and 7 being extremely rapid^19^. Accordingly, the landslide that’s occurring in Doi Chang can be categorized between Class 1 (1.31 mm/month) and 2 (131 mm/month) and are described as extremely slow and very slow landslides. Another classification of landslide is referred to as “creep” which is considered to be imperceptible. Creep is a continuous movement that proceeds at an average rate of less than 30 cm per decade, equivalent to 2.54 mm/month^2^.

**Figure 4:**
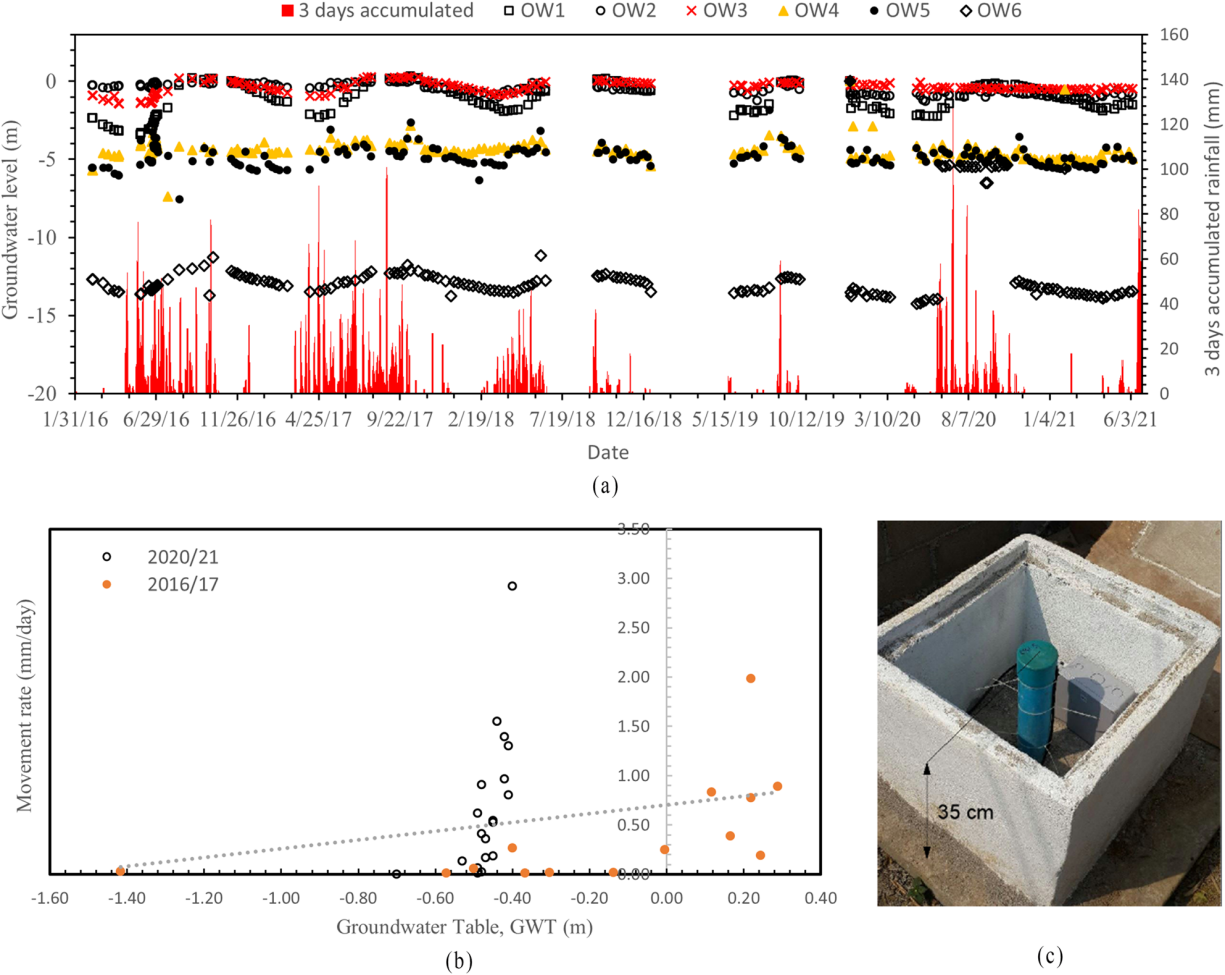
(**a**) Groundwater level readings recorded for a period of 4 years (**b**) Relationship between groundwater level, rainfall and movement rate (**c**) Observation well tube extended above ground level.

From the data observed through inclinometer and GPS survey, it can be seen that the occurring slope movement can either be categorized as creep or in between Class 1 and 2. The severity of landslide considering the velocity of slope movement is not high, however the velocity of slope movement lying in between Class 1 and 2 can bring in structural damage as evident in the study. Such variations in velocity of slope movement are a function of seasonality, bringing changes in level of groundwater table as depicted in Fig. 4a,b.

Figure 4a presents the record of 3 days accumulated rainfall with changing groundwater table for more than 5 years period (2016–2021 AD). A positive correlation between the rainfall and the groundwater levels exists, meaning with increase in rainfall there is increase in groundwater table and vice versa. However, as discussed in Sect. 2, the topography of the hill slope was such that it led to the concentration of water in a particular area. The flowing surface water would get concentrated in a place where OW3 was installed, because of which the water levels in OW3 remained nearly at the ground level.

Figure 4b is a plot of movement rate with the changing groundwater table from OW3. As the groundwater level increased, the rate of movement increased along with it. The movement rate is calculated from the inclinometer readings for the nearby ground surface for the period of 2016/17 and 2020/21. It is observed that the movement rate increased with an increase in groundwater levels. It is to be noted that GWT readings at some instances are above zero values because the observation well tubes were extended above ground for 35 cm as shown in Fig. 4c. It is also to be noted that the groundwater level reading for 2020/21 did not change remarkably as it is suspected that the observation wells were damaged due to ground movement as indicated by the inclinometers.

## Field and laboratory tests

Several field and laboratory tests were performed to determine the properties of the soil and to explore the subsoil stratum. Four test pits were dug to obtain the disturbed soil samples and were used for different tests like sieve analysis, specific gravity, Atterberg’s limit, permeability test. In the site, a resistivity test was performed to determine the nature of the underlying soil surface. Similarly, SPT test was also performed at the site to confirm the results of resistivity test and to assess the nature of the underlying soil rock. ERT and SPT tests conducted confirmed the presence of granular materials spread over a large area as observed from the test pits.

In the laboratory, the residual shear strength of the soil was determined using a cut plane method. The obtained soil strength parameters were then used for the slope stability analysis.

### Basic soil tests

A comprehensive soil testing was performed to determine the physical properties of the soil. The results of Atterberg’s limit test, natural water content test, specific gravity, and permeability test are summarized in Table 1.

**Table 1:** Index properties of the soil.

Physical properties of soil	No. of samples tested	Mean	Coefficient of variance (COV)	Range
Liquid limit, LL (%)	53	47.1	15.63	35.80–62.62
Plastic limit, PL (%)	53	33.33	14.44	24.89–44.25
Plasticity index, PI (%)	53	13.77	29.41	5.74–25.16
Flow index	53	13.84	22.70	7.81–24.87
Specific gravity of soil	53	2.65	3.59	2.44–2.83
Permeability (cm/s)	3	8.28 ×10^–5^	–	6.09 × 10^–6^–10.60 × 10^–4^
Natural water content (%)	23	34.42	22.78	23.09–45.61
Dry unit weight (kN/m^3^)	23	1.21	12.92	11.0–12.0

From the 53 samples testes, the average liquid limit was about 47%, with COV of 15.63. Similarly, the mean plasticity index of the soil is 13.77%, and COV is 29.41. It indicates that the soil is medium plastic in nature. The specific gravity of the soil was 2.65. The mean value of the flow index is 13.84 suggesting the high potential of the soil to change its state from plastic to liquid. The permeability obtained through the constant head^20^ method showed an average value of 8.28 × 10^–5^ cm/s indicating the semi-permeable nature of the deposit. The natural water contents of the soils were examined on the samples collected during the rainy season at a depth of 1–2 m and the average was around 34.5% indicating the likely plastic state of the soil in the remoulded condition. The dry and wet unit weights were found to be on the average of 12.1 and 15.9 kN/m^3^, respectively which also shows the high content of water in the soil.

**Figure. 5:**
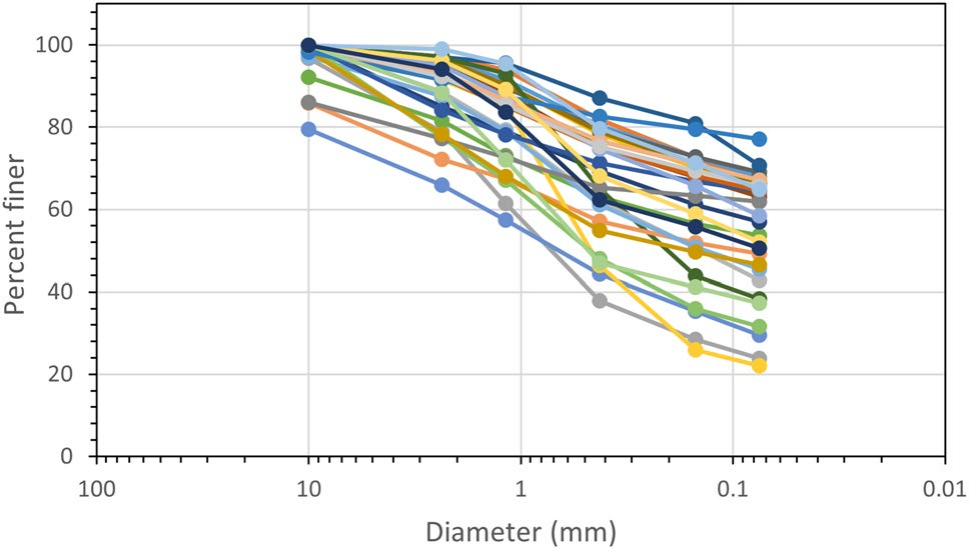
Grain size distribution curve.

Similarly, sieve analysis was performed on the disturbed samples obtained from the test pits. The result of the sieve analysis is given in Fig. 5. The majority of the soil portion is fine-grained soil, and using a plasticity chart the soil can be classified using the USCS system. It was found that the soil at the top surface consisted of ML type (silt with low plasticity) whereas the soil at a depth of 3 m consisted of MH type (silt with high plasticity).

### ERT and SPT test

**Figure. 6:**
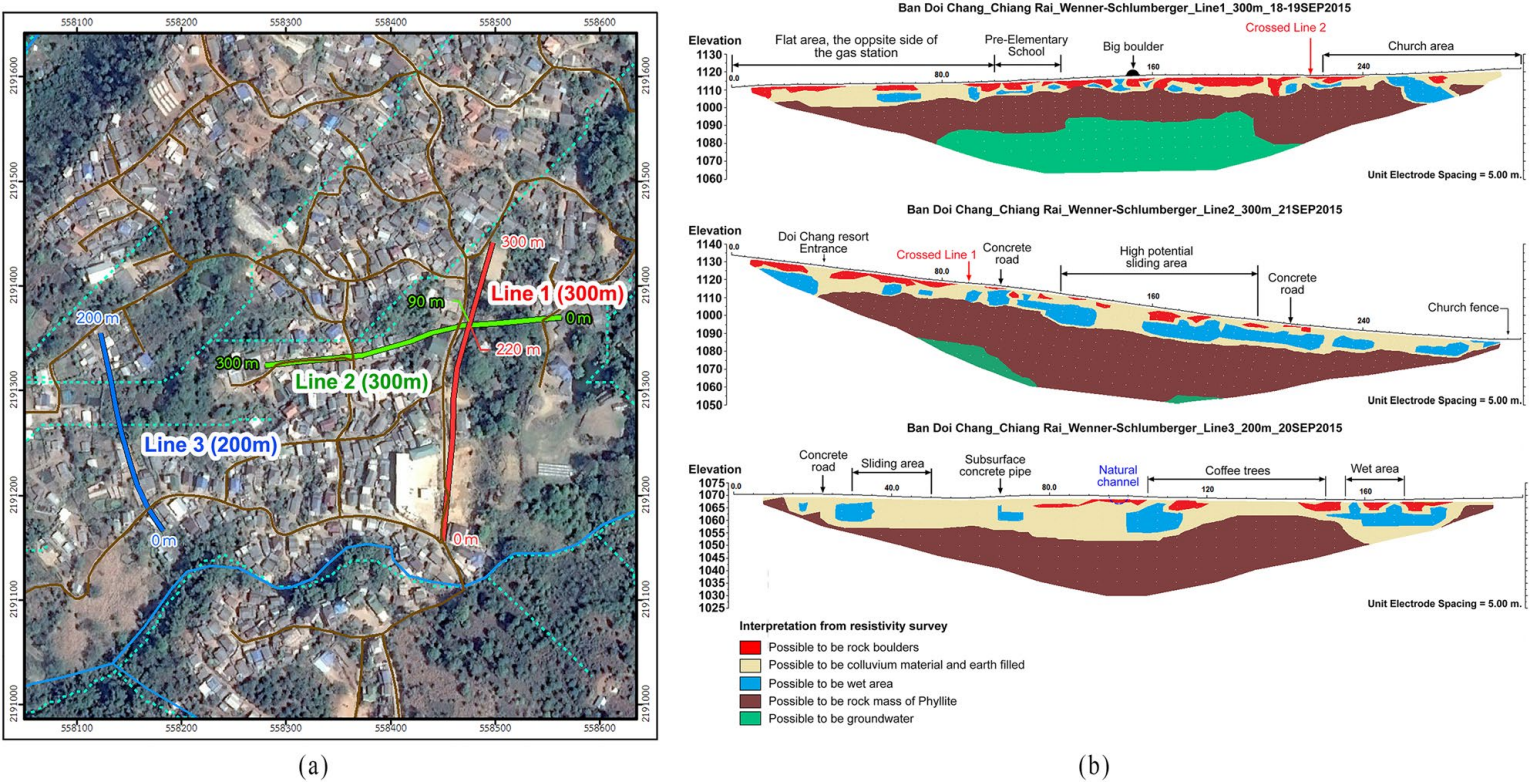
(**a**) Lines of resistivity survey (**b**) Results of resistivity survey.

Among many in-situ geophysical techniques that provides information on greater volume of soil, ERT test has been widely applied for landslide investigation^21–23^. In order to determine the nature of the subsoil layer, ERT test was performed and was supplemented with the SPT test. The ERT test was carried out along three lines, namely Lines 1, 2, and 3 (Fig. 6) of the study area. The lines are 300 m, 300 m, and 200 m in length, respectively. Wenner-Schlumberger^24^ type of electrode arrays arrangement was used, and Res2Dinv^25^ software was used for the electrical resistivity data inversion.

The interpretation of the electrical resistivity shows that there are three main layers of the geomaterials covering the site. The thickness of the first layer was found to be varying from 7 to 32 m, consisting of colluvium soil. Additionally, dispersed water pockets were also observed with a low electrical resistance of 4–16 Ω-m. The second layer possibly consisted of the phyllite rock mass as the electrical resistance of 64–1024 Ω-m was observed. The results of the lower depth were varying in different lines of the test conducted. In Line 1, at the lower depth, possible groundwater was observed, and in Line 3, the resistance offered by the soil indicated no observation of groundwater level.

**Figure. 7:**
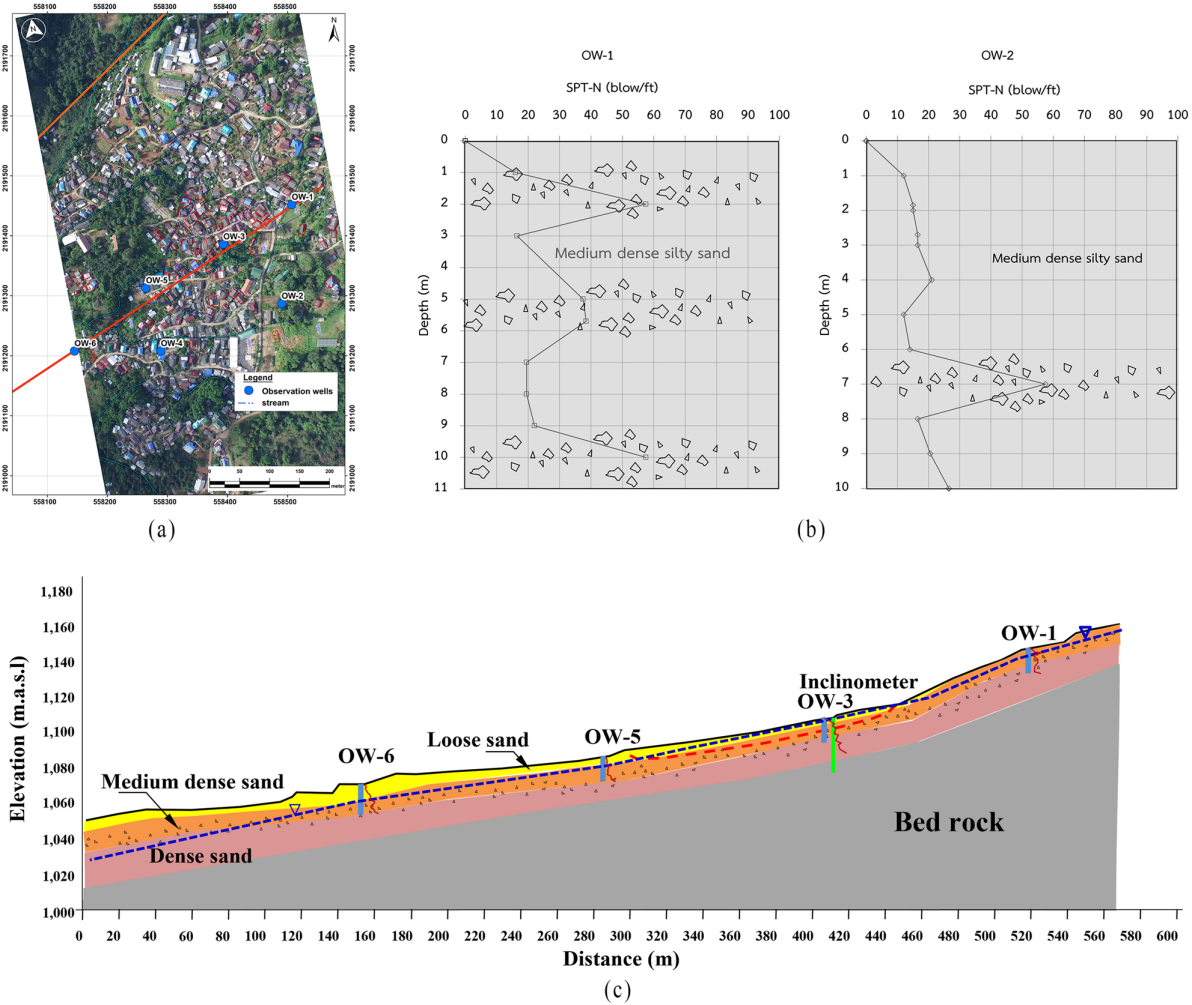
(**a**) Alignment passing through the damaged section (**b**) Example of SPT test results at OW1 and OW2 demonstrating fragments of boulder (**c**) Transverse section of the lithology of the slope.

SPT test^26^ was carried out at the location of six observation wells (Fig. 7a). For reference, the results of the SPT test obtained at OW1 and OW2 are given in Fig. 7b. It can be seen that the subsoil basically consisted of medium dense silty sand with fragments of boulders spreading out along the depths. The SPT-N value rose high to 50 whenever it hit the boulder layer, whereas, for the medium dense silty sand, the SPT-N value is at an average of 15. A transverse section of a straight alignment passing through the damaged area was developed based on results of SPT and resistivity test (Fig. 7c). The subsoil basically consisted of medium dense sand intertwined with boulders. Hence, the presence of colluvium deposits was confirmed from test pits, SPT test, and resistivity test.

### Residual shear strength test

Landslides involve a large strain shearing of soil, and hence residual shear strength of the soil is an appropriate shear strength value to evaluate the slope stability. In the residual state, the soil undergoes a large strain, the interparticle bonds are broken, and soil particles are oriented parallel to the slip plane. In^27,28^ have successfully applied the residual shear strength for slope stability analysis of reactivated landslides. In this study, the residual strength of the soil were obtained using unconventional testing approach (not included in ASTM standard) a cut-plane method^29^.

In this method, the sample was loaded in a direct shear box of 63 mm diameter and pre-shear plane was defined prior to the testing. The initial void ratio of the sample was found to be 1.51. The initial moisture content of the sample was obtained and the target degree of saturation was achievied by spraying water using a very fine spray and measuring the weight of the added water. The degree of saturation was then calculated using Eqs. (1) and (2),

1 $$e= \left(\frac{{G}_{s}(1+w)}{{\gamma }_{s}}\right).{\gamma }_{w}-1$$

2 $$Sr= \frac{w.{G}_{s}}{e}$$

where, *e* is the void ratio, *G*_*s*_ is the specific gravity of soil, $w$ is the water content of the sample, ${\gamma }_{w}$ is the unit weight of water, ${\gamma }_{s}$ is the unit weight of the soil and $Sr$ is the degree of saturation of the soil sample.

The degree of saturation was maintained at 100% as far as possible and during shearing, soil samples were wrapped with thin plastic cover to maintain the moisture content of the soil sample. The sample was sheared at the rate of 0.01 mm/min to prevent the development of pore water pressure (drained test).

From the cut-plane direct shear test, the residual cohesion of colluvium soil (*c*_*r*_) was observed to be 7.208 kPa and the residual friction angle of colluvium soil (${\varphi }_{r}$) as 21.97 degrees. According to^27,28^, the residual state would be similar to the critical state shearing for soils with low clay content, while the residual condition would be of lower resistance if the soil has high clay content. It is observed that samples demonstrated cohesion intercept and is thought to be from the effect of various factors such as suction, bonding, and the size effect of the shear box.

## Landslide susceptibility assessment

A physically based model has been adopted among different methods of landslide susceptibility assessment like knowledge-driven approach and data-driven methods. Considering geometrical and geotechnical characteristics, a physical slope model called infinite slope model^30^ has been used for slope stability analysis. Physically based models are widely used as they offer better predictive capability with quantitative assessment of the effects of individual parameters^31^.

Furthermore, infinite slope stability offers flexibility to locate the changing water table at the required depth below the ground surface; hence effect of changing groundwater table on the stability of a slope can be observed. The ratio between the depth of the failure plane and the length of the failure zone is about 1.6%; hence the landslide is considered shallow (top few meters) and parallel to the ground surface. In rainfall-induced landslides, the failure surface is often shallow and this model has been used to analyse susceptibility in many of the previous studies^32–38^. Moreover, the model has also been used to calculate FS for shallow landslides in colluvium deposit^39^, and thus infinite slope model was appropriate for the current study. Based on the model, Eq. (3) gives the FS for the stability analysis of slope inclined at an angle $\beta$.

3 $$FS=\frac{{c}^{^{\prime}}\cdot \mathit{sec}\beta +\mathit{cos}\beta \cdot \left({\gamma }_{sat}z-{\gamma }_{w}h\right)\cdot {\mathit{tan}\phi }^{^{\prime}}}{z\cdot {\gamma }_{sat}\cdot \mathit{sin}\beta }$$

**Figure. 8:**
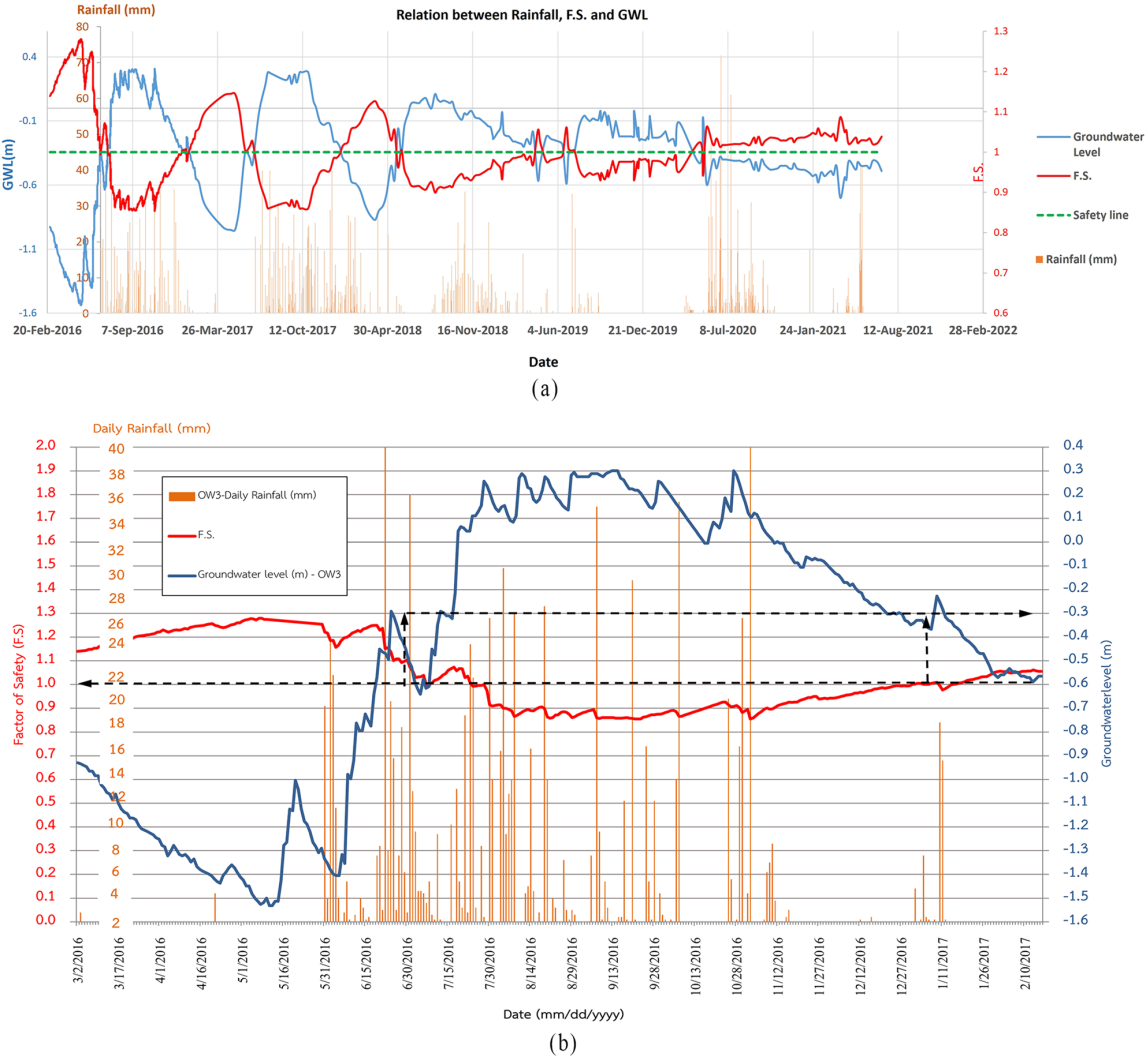
(**a**) Relation between rainfall intensity, FS and groundwater table for a period of 4 years (**b**) Zoomed-in section for 2016.

In above, *c’* is the effective soil cohesion in kPa, $\gamma_{sat}$ is the saturated soil unit weight in kN/m^3^, $\gamma_{w}$ is the unit weight of water in kN/m^3^, *h* is the height of the water table in m and $\phi^{\prime}$ is the effective friction angle in degrees. Residual cohesion value (C_r_) of 7.21 kPa and friction angle (${\phi }_{r})$ of ${21.97}^{o}$ were used in above equation obtained from the residual strength test. Similarly, the inclination of the slope ($\beta$) as 10°, depth ($z$) of failure plane as 5 m, ${\gamma }_{sat}$ as 1.8 t/m^3^ were used in the above equation. The level of groundwater level ($h$) was varied according to the data recorded from the observation well (OW3) and a plot of FS along with rainfall and groundwater level recorded over a period of nearly four years is given in Fig. 8a.

The FS value of 1 has been kept as a safe value indicated by a green dotted line. It can be seen that the FS decreased below 1 as the groundwater level rose to level of 0.3 cm from the ground surface. The safety factor less than unity (FS < 1) reflects the influence of ground water rise on reduction in shear resistance and increase in landslide velocity. According to^40^, the calculated safety factor of 1 refers to the static equilibrium at the onset of movement when the resisting force just balances the driving force. As groundwater level begins to rise further, the shear resistance decreases, in response to reduction in effective stress, while the driving force remain practically constant, resulting in increase in landslide velocity. Some form of dynamic resistance would compensate for the reduction in static resistance. The consequence is that the calculated static safety factor falls below the threshold level of 1 during the accelerated movement.

The increase and decrease in groundwater level is in sync with the rainfall. Figure 8b shows the zoomed in section for the relationship between rainfall, groundwater table and FS for 2016. The FS was above 1 from 2nd March to 29th June (dry season) but it can be seen that it started decreasing once the rainfall began from 30th May and remained below 1 from 19th July to 18th Dec 2016 (wet season). The groundwater table also maintained a depth of 0.3 cm throughout that period.

**Figure. 9:**
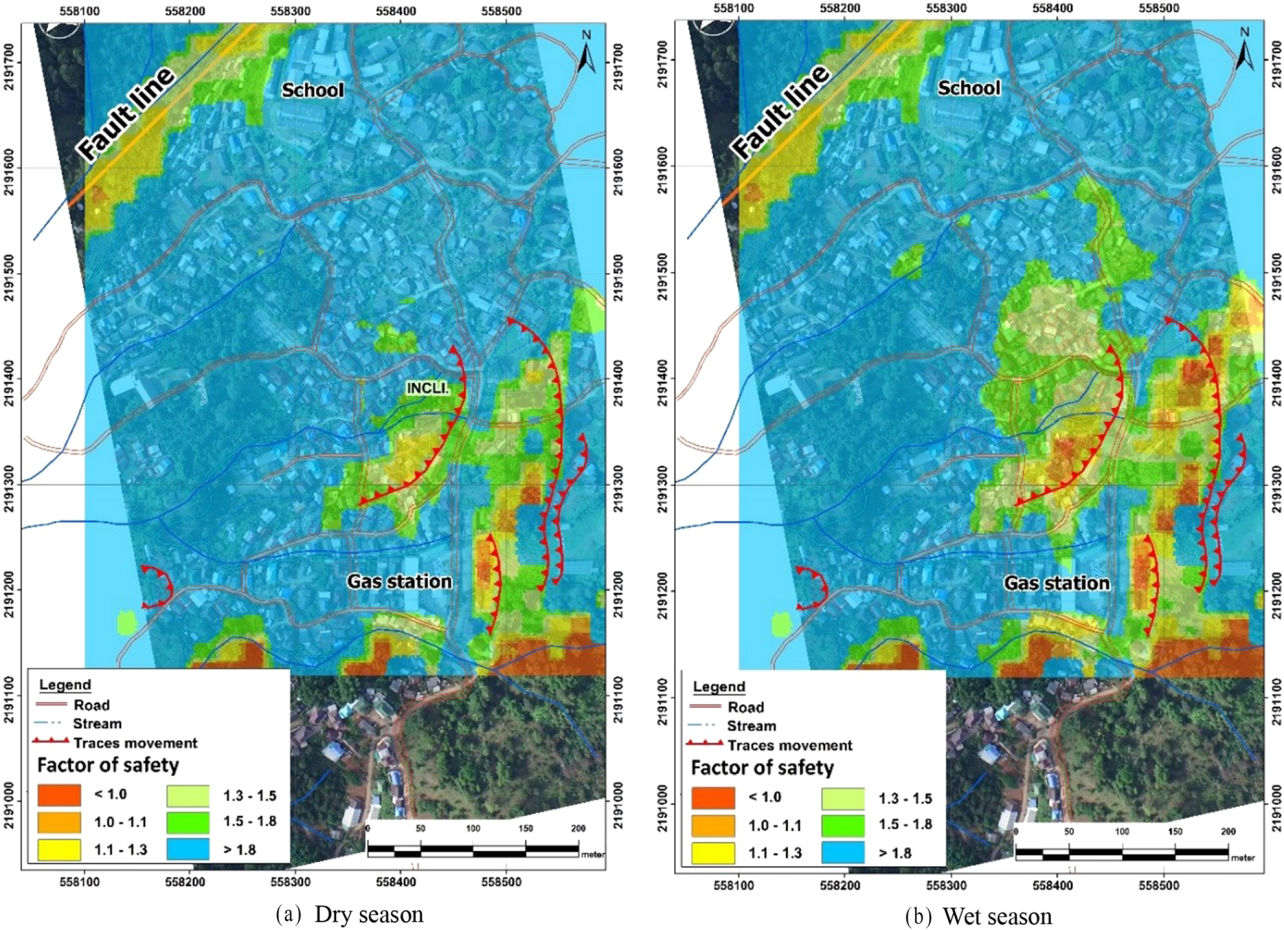
Factor of safety for dry and wet season.

The use of physical model along with GIS methods enables slope stability analysis over a large area. For this purpose, ArcGIS was used to show the results of risk assessment for landslides. The necessary information like slope gradients are derived from a digital elevation model (DEM) while the geotechnical parameters used in the infinite plane slope stability model like angle of internal friction (ø = 21.97°), soil density (17.65 kN/m^3^) and cohesion (7.21 kPa) are obtained from the aforementioned tests. The DEM used for the model was derived by digitizing contour lines from topographic maps at the 1:50,000 scale with a vertical interval of 5 m. The DEM prepared in the ArcGIS software was refined by adding elevation points extracted from the processing of aerial photographs. Figure 9 shows the results of change in FS during dry and wet seasons.

The above assessment shows that the FS increased with a decrease in the groundwater table. Hence, one of the appropriate measures to control the slope movement can be through horizontal drains. Such remedial measure has been proven effective in colluvium deposit^41^. Identifying proper mitigation plan can be another study for which the result from the current research is helpful. The results from this study, therefore, are being used by the officials in the ministry level.

## Conclusion

The slope movement in the colluvium deposit of Doi Chang, Thailand, was extensively studied using in-situ ground-based monitoring techniques. The paper discussed the slow slope movement which caused recurrent damage to the structures. The paper also evaluated its FS using the infinite slope model. Summarization of the results could explain the relationship between the rainfall, groundwater level and the slope movement. Based on the results, the following conclusions can be made:

1. Extensive site investigation and in-situ tests like ERT and SPT confirmed colluvium deposits along the depths in the middle portion of the hill.
2. While mapping the damaged buildings, those categorized under major damages were lying in the area where the rainwater gets concentrated. The inclinometers installed around that area demonstrated shear plane at a depth of 3–5 m.
3. The rate of movement observed from the inclinometers and GPS can be categorized as creep (below 2.54 mm/month) or in between classes 1 and 2. The creep rate was observed during the dry seasons, while it was observed to get as high as 66 mm/month during the rainy season. Such rate of movement did not cause catastrophic failure of the slope, but it was sufficient to cause structural damages. Furthermore, the direction of the slope movement was determined by GPS stations allocated over larger areas. The direction was to the southwest of the village, forming the downslope of the area.
4. The plot of groundwater levels (OW1–OW6) with the 3 days accumulated rainfall suggested a positive correlation. The observation well, OW3, installed at the water concentrating area showed consistently higher readings compared to other observation wells.
5. Residual shear strength parameters obtained from the cut plane method was used to evaluate the FS from the infinite slope model. The FS fell below 1 during the rainy season as the groundwater table rose to 0.3 m, proving that the rise in the groundwater table had a significant role in the slope movement under the colluvium deposit.
6. Through the integration of ArcGIS with the infinite slope model, a map depicting the change in FS during the dry and wet season was developed for a larger area. The map showed that the regions where FS fell below 1 expanded during the wet season, thereby delineating the risky areas.

The identification of the causes for the slope movement through the research has raised awareness and informed community. Though catastrophic failure of the slope is unexpected, people are still suffering from the recurrent damage of the structures. Mitigation plan therefore are important, and the research has helped the officials in the ministry level for setting it up.
